# The ecological function of thyroid hormones

**DOI:** 10.1098/rstb.2022.0511

**Published:** 2024-03-25

**Authors:** Jann Zwahlen, Emma Gairin, Stefano Vianello, Manon Mercader, Natacha Roux, Vincent Laudet

**Affiliations:** ^1^ Marine Eco-Evo-Devo Unit, Okinawa Institute of Science and Technology, Onna, Okinawa 904-0495, Japan; ^2^ Computational Neuroethology Unit, Okinawa Institute of Science and Technology, Onna, Okinawa 904-0495, Japan; ^3^ Marine Research Station, Institute of Cellular and Organismic Biology (ICOB), Academia Sinica, Taipei, Lan 262, Taiwan

**Keywords:** thyroid hormones, ecology, hormone function, adaptation

## Abstract

Thyroid hormones (TH) are central hormonal regulators, orchestrating gene expression and complex biological processes vital for growth and reproduction in variable environments by triggering specific developmental processes in response to external cues. TH serve distinct roles in different species: inducing metamorphosis in amphibians or teleost fishes, governing metabolic processes in mammals, and acting as effectors of seasonality. These multifaceted roles raise questions about the underlying mechanisms of TH action. Recent evidence suggests a shared ecological role of TH across vertebrates, potentially extending to a significant portion of bilaterian species. According to this model, TH ensure that ontogenetic transitions align with environmental conditions, particularly in terms of energy expenditure, helping animals to match their ontogenetic transition with available resources. This alignment spans post-embryonic developmental transitions common to all vertebrates and more subtle adjustments during seasonal changes. The underlying logic of TH function is to synchronize transitions with the environment. This review briefly outlines the fundamental mechanisms of thyroid signalling and shows various ways in which animals use this hormonal system in natural environments. Lastly, we propose a model linking TH signalling, environmental conditions, ontogenetic trajectory and metabolism.

This article is part of the theme issue ‘Endocrine responses to environmental variation: conceptual approaches and recent developments’.

## 1. Introduction

Thyroid hormones (TH) play a pivotal regulatory role enabling synchronized transformations across diverse tissues during post-embryonic development as well as homeostatic regulation of metabolism and energy expenditure. TH orchestrate intricate biological processes, allowing growth and reproduction to occur in various environments. By acting on target cells and tissues, they influence gene expression and cellular responses, which control molecular cascades that fine tune developmental pathways and responses to external stimuli [1,2].

Overall, TH govern three very different processes in vertebrates. In amphibians and teleost fishes, they trigger metamorphosis, a remarkable post-embryonic transition through which larvae transform into juveniles (e.g. anuran tadpole to froglet transition). In these species, a peak of TH triggers and coordinates the whole transformation, which is essential, at least in frogs, to begin the transformation [2]. Secondly, in mammals, including humans, TH regulate essential homeostatic processes such as basal metabolism, thermogenesis, and heartbeat. Often, such regulatory roles result in increased energy expenditure, as demonstrated by increased oxygen consumption following TH treatment [3]. The significance of this role is further highlighted by the numerous thyroid axis-related diseases that can occur. Thus, TH levels must be tightly regulated in adult mammals, as excessively low or high TH levels can have detrimental effects. Accordingly, a negative feedback system allows TH to regulate their own levels through the hypothalamo-pituitary-thyroid (HPT) axis. Thirdly, TH act as major effectors of seasonality, the process by which species adapt their physiology and reproduction to annual changes in temperature and photoperiod [4]. In that case, precise local regulation of TH production in the hypothalamus and pituitary transform daylight length and thermic information into endocrine and genomic regulation.

**Figure 1.**
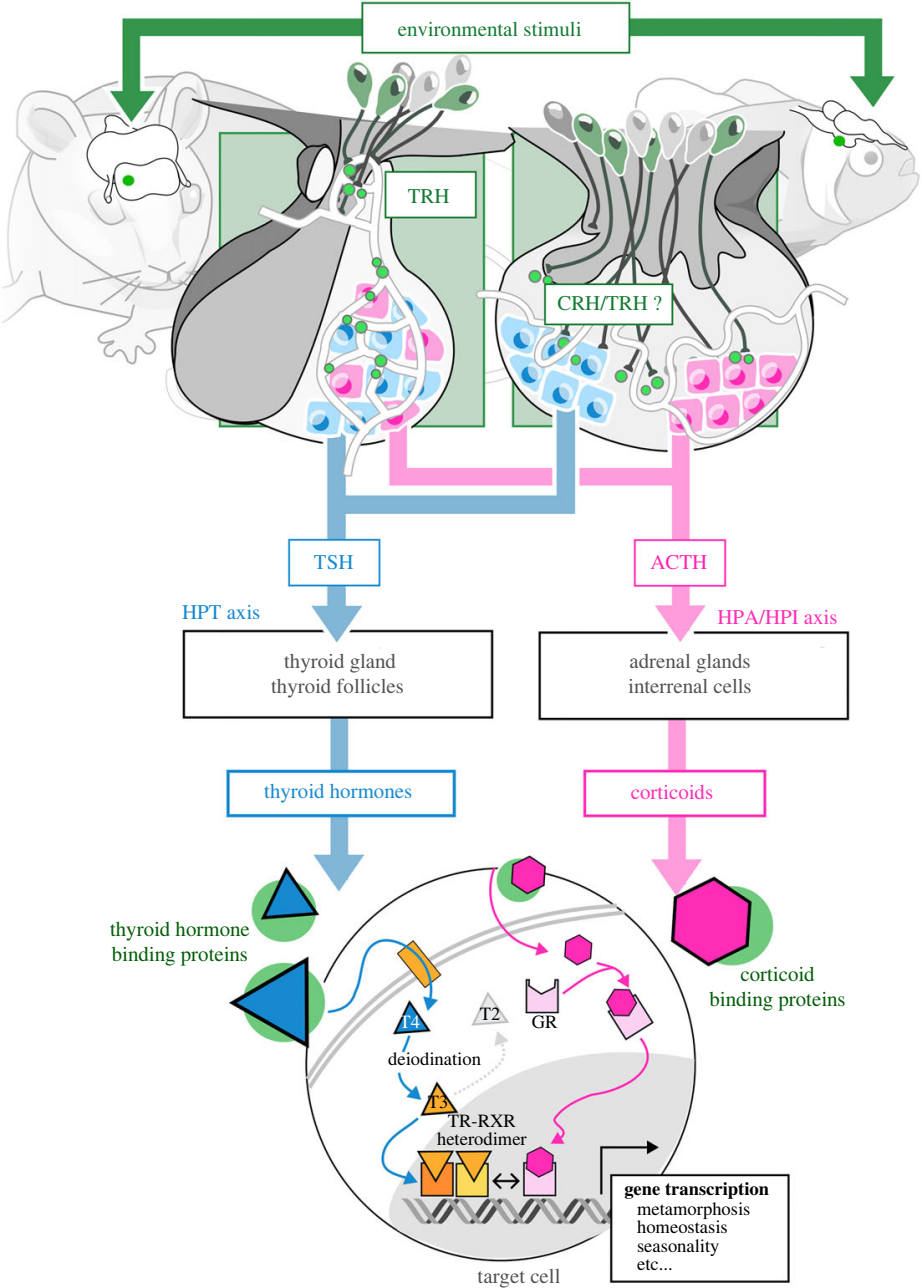
Summary diagram of the hypothalamo pituitary thyroid axis (HPT; light blue), and hypothalamo pituitary adrenal/interrenal axis (HPA/HPI; pink) in vertebrates. Special emphasis is put on the differences between the mode of hypotalamic peptide delivery to the adenohyphysis (green inset, light grey lobe) in mammals (left) versus fishes (right). See text for details. TRH: thyrotropin-releasing hormone; CRH: corticotropin releasing hormone; TSH: thyroid-stimulating hormone; ACTH: adrenocorticotropic hormone; GR: glucocorticoid receptor; TR: thyroid hormone receptor, RXR: retinoid X receptor.

The marked difference between TH roles across vertebrate species has long remained an enigma. It may be attributed to historical bias in the model systems used for TH research—frogs for metamorphosis and human/mouse for metabolic studies. It is also clearly possible that there has been strong evolutionary diversification of TH function across vertebrates. Yet, all three roles could alternatively just be versions of a same basic function with different manifestations in different organisms. Recent findings reinforce the latter hypothesis, which we will develop in this review by suggesting a shared ecological role of TH across vertebrates, potentially extending to a significant portion of bilaterian species. In fact, we believe that on top of regulating acclimation to different environments, TH govern ontogenetic transitions by ensuring their alignment with the surrounding environment, particularly with regards to energy expenditure, which must indeed correspond to available resources. These transitions occur during post-embryonic development (i.e. metamorphosis, which is present in all vertebrates); yet there are also other transitions, such as seasonal ones, during which it is important to adjust physiology to changing resource availability. The operating logic, at least common to all vertebrates, is therefore to orchestrate transitions and to ensure that they are done in harmony with the environment in which they occur. After briefly outlining the fundamental mechanisms of thyroid signalling (figure 1), we will explore the role of TH in natural environments. While achieving comprehensiveness is not feasible, this overview will provide a broad perspective on how animals use this hormonal system before concluding with the presentation of an overarching model.

## 2. Vertebrate thyroid hormone signalling in a nutshell

In vertebrates, the TH signalling pathway starts in the hypothalamus, where environmental and endogenous cues trigger the release of thyrotropin releasing hormone (TRH) into the hypophyseal portal system, a microcirculatory system ensuring that the hypothalamic peptide reaches the anterior pituitary at the right concentration (figure 1). There, TRH binds to its receptors, which in turn stimulate the production and release of thyroid-stimulating hormone (TSH) in the blood circulatory system. It is important to note that thyroid axis regulation by TRH only occurs in mammals, adult frogs, and adult teleost fishes (see for example [5]). During metamorphosis in frogs, and probably in fishes, TH control is instead coupled to the corticotropin-releasing hormone (CRH) and the stress axis [6].

TSH produced by the pituitary stimulates the production of TH by the thyrocytes of the thyroid gland (or follicles in teleosts). Accordingly, TSH upregulates the sodium-iodide symporter that allows iodine entry in thyrocytes, as well as thyroid peroxidase that stimulates the fixation of iodine to thyroglobulin, the precursor of TH, whose gene expression is also stimulated. The release of TH into the blood circulation is also activated by TSH. TH levels are finely regulated by a negative feedback mechanism by which TH downregulate both TRH and TSH expression [1]. This regulation of TH signalling most probably occurs in all vertebrates, although there is a strong bias of data availability in mammals and amphibians. These regulations must be studied in more detail in other vertebrates.

The thyroid gland produces mostly 3,5,3′,5′-tetraiodothyronine (also called thyroxine or T4) into the bloodstream. T4 can be transformed by deiodination into T3 (3,5,3′-triiodothyronine), the active compound that binds to its specific receptors. Deiodination is ensured by deiodinases which remove iodine from T4 and T3, hence finely regulating the amount of active hormone in target tissues. Deiodinase 2 (dio2) activates T4 into T3, whereas deiodinase 3 (dio3) transforms T3 into T2, which is much less active, and is therefore associated with decreased TH signalling. The third deiodinase, dio1, can either activate or degrade TH, making its presence in tissues more complex to decipher (reviewed in [7]).

In the bloodstream, TH are carried by specific transporters to target tissues. Inside target cells, T3 binds to the thyroid hormone receptors (TR) which, with their heterodimeric partners, retinoid X receptors (RXR), are involved in the regulation of a multitude of target genes [1,8]. Furthermore, TH can also control cellular changes via nongenomic mechanisms, e.g. by acting on membrane receptors, transporter molecules, and cytoplasmic receptors, and by altering cellular pathways (e.g. cyclic adenosine monophosphate, inositol triphosphate, Ca^2+^), indirectly modifying gene expression [9,10].

Interestingly, TH signalling is also tightly linked to other endocrine systems, in particular glucocorticoids (GC) that control stress and metabolism. GC synthesis is regulated by the hypothalamic CRH which controls the production of adrenocorticotropic hormone (ACTH) in the pituitary. ACTH then stimulates GC synthesis by the interrenal gland. The interplay between GC and TH occurs at multiple levels, including gene regulation and hormonal status [6]. For instance, in mammals, the activation of the stress axis results in decreased TH signalling, reduced TSH excretion from the anterior pituitary, and reduced (TH) production from the thyroid gland [11,12]. Another key cross-talk between TH and GC is the one observed during amphibian metamorphosis. Indeed, external stimuli such as decreased water level, increased density, and the presence of predators can trigger tadpole metamorphosis by inducing both TH and GC synthesis. However, we still lack a comprehensive overview of such interactions, mostly because of the lack of diversity in experimental models. It is absolutely clear, however, that TH are not the only players in the physiological and ontogenetic transitions discussed here, and that GC has a major role both on its own and via its interactions with TH.

## 3. Thyroid hormones and ecology

### (a) Resource availability and energy expenditure

The importance of TH for metabolic regulation has long been known and is well illustrated in mammals. TH metabolic action takes place in brown adipose tissue (see the next section, ‘thermogenesis’), but also in white adipose tissue, in muscles, and in the liver, where it stimulates mitochondrial metabolism [3,13]. Specifically, TH accelerate both the citric acid cycle and oxidative phosphorylation in mitochondria, inducing ATP production and subsequent energy consumption. TH is also involved in both glycolysis and lipid metabolism regulation, confirming its pivotal role in metabolic homeostasis. Most experiments investigating the metabolic role of TH in mice have been driven by the ongoing epidemic of obesity and metabolic disorders in humans. This epidemic is characterized by excess food consumption and reduced physical activity, which lead to heightened fat storage, linking the TH pathway with both resource availability and energy expenditure.

Generally, the metabolism of wild animals is adapted to scarce resources. Several lines of evidence have correlated TH levels with food availability in terrestrial mammals such as rodents, carnivores, primates, and ungulates, among others [11]. Classically, TH levels decrease during periods of energy restriction and increase when energy is abundant [11]. For example, the mantled howler monkey (*Alouatta palliata*) is a frugivore-folivore whose TH levels are positively and negatively correlated with fruit and leaf intake, respectively [14]. The decrease in TH levels attributed to higher leaf consumption was associated with an energy-saving strategy during times of fruit scarcity.

Another interesting example is the possible link between energy deficit, TH, and stress in racing horses. Indeed, inexperienced horses do not feed during transport and produce lower levels of TH and higher levels of cortisol [15]. The link between changes in energy levels and TH levels is evident and has been confirmed across various species and contexts, yet our current understanding is limited to adult organisms [11]. It is also important to highlight that many of these studies solely quantify TH levels. Consequently, we possess little information about the actual activity of the TH signalling pathway or about the underlying mechanisms in these scenarios.

**Figure 2.**
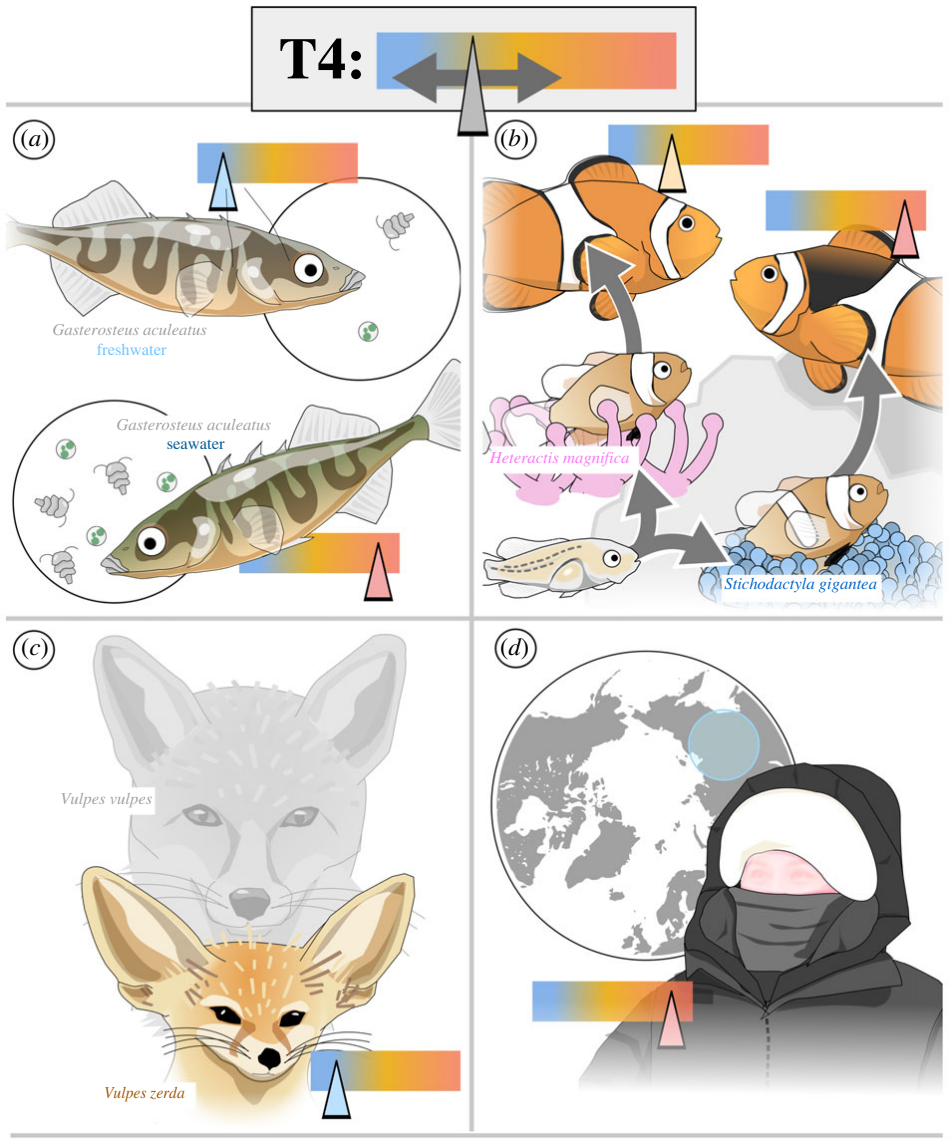
Tuning of metabolism, energy demands, and environment through the modulation of TH levels: illustration of some of the main case-studies mentioned in the text. Differences in TH levels across species or populations have been found to tune organisms to: differences in food availability across environments (top left, G*asterosteus aculeatus*); differences in sea anemone hosts (top right, *Amphiprion percula*); adaptation to high temperatures (bottom left, *Vulpes zerda*); and adaptation to cold temperatures (bottom right). Tuned TH levels are here represented by a slider dial (left, low; right, high).

The link between energy expenditure and TH is not restricted to mammals but is also found in teleost fishes. Three-spine sticklebacks (*Gasterosteus aculeatus*) are marine fish that have been repeatedly and convergently isolated in freshwater, a relatively energy-poor environment compared to the marine realm [16] (figure 2*a*). Fish living in energy-rich marine environments have higher TH levels than populations in nearby freshwater streams, and these differences are maintained in populations living in captivity, suggesting a genetic basis [17,18]. Indeed, the authors observed that *TSHβ2*, a gene controlling T4 production, had a lower expression level in sticklebacks from freshwater ecotypes than compared to their marine counterparts, which was coupled with differences in its regulatory region. This suggests a direct connection between the feedback mechanisms governing T4 production and freshwater ecotypes, with a lowered *TSHβ2* expression and lower TH levels. This reduction is associated with decreased energy expenditure ([18], reviewed in [19]).

Additionally, we have shown differences in TH levels between distinct ecotypes of anemonefish, which are associated with metabolic differences, although we still do not know if there is a link with energy expenditure [20,21]. The anemonefish *Amphiprion percula* can live in association with different species of sea anemones (figure 2*b*). New recruits settling in the carpet sea anemone *Stichodactyla gigantea* form their white bars more rapidly. They also have higher TH level and higher expression of *duox*, a critically important gene for TH production whose knock-out induces hypothyroidy in both zebrafish and humans [20]. Furthermore, glycolytic genes, as well as genes associated with the citric acid cycle and β-oxidation, had higher expression levels in young juveniles in *St. gigantea*. This elevated gene expression is in accordance with the metabolic shift observed in these animals during metamorphosis and suggests that these fish, which will remain in the same host throughout their life, use the TH system to fine-tune their physiological regulation depending on their host. However, unlike sticklebacks, this regulation of TH production does not translate into a genetic difference, as individuals from the same population can settle in different sea anemones (i.e. it is highly plastic).

Overall, when facing higher energy demands, vertebrates can activate their TH signalling to facilitate resource mobilization when the necessary resources are available. Conversely, when resources are limited, TH signalling can be reduced to lower energy expenditure and ensure that the needs of the organism remain aligned to the available resources [22]. The main role of TH is precisely to achieve this coupling between resources and metabolism while ensuring that the coordination of the metabolic response to available resources is well integrated at all levels.

### (b) Thermal adaptation

In homeothermic organisms, the regulation of body temperature (thermogenesis) is intrinsically linked to metabolism and energy expenditure. Maintaining constant body temperature is energetically demanding and it is therefore not surprising that TH are a major player in the fine regulation of thermogenesis [11,23,24]. Primarily, TH enhance the basal metabolic rate, leading to increased energy expenditure and heat production during the body's resting state by driving mitochondrial activity. The subsequent engagement of the sympathetic nervous system by TH triggers the release of norepinephrine, a driver of thermogenesis. This prompts brown adipose tissue activation, which fuels heat production through enhanced lipolysis and subsequent energy combustion.

Adaptation to environments with extreme thermic conditions provides valuable information on the role that TH play in thermoregulation in wild animals. In deserts, animals need to reduce their metabolic rate to avoid overheating and cope with food rarity [25]. Studies using many different vertebrates have shown that this is achieved at least in part by reducing TH levels. This is the case in the kangaroo rat (*Dipodomys merriami*) and Libyan gerbil (*Meriones libycus*), in which it was further shown that manipulating TH levels affects metabolic rate [26,27]. Of particular interest is the recent genome analysis of two desert adapted carnivores, the fennec (*Vulpes zerda*) and Rueppell's fox (*Vulpes rueppelli*), that show numerous signatures of adaptation, including in TH signalling genes [28] (figure 2*c*). In Rueppell's fox, SLCO4A1, a solute carrier protein that can transport T4 is under positive selection, and a sulfotransferase implicated in TH metabolism showed a signature of selection. In accordance with these data, these two foxes displayed significantly lower levels of T4 than the African red fox (*Vulpes vulpes*) did [28]. It is very clear from all these cases that thermal regulation is intimately linked to metabolic activity.

What is true for very warm conditions is also true for extremely cold environments, in which TH are also an important player. In contrast to a hot desertic condition, metabolic rate should be increased to produce heat under cold conditions, leading to an increase in TH levels (reviewed in [29]). One of the most fascinating examples comes from human populations. Physiological studies of various indigenous Siberian groups have revealed several traits that reflect underlying genetic adaptations to their cold environment (figure 2*d*). More specifically, an elevated basal metabolic rate and an increased level of T4 compared with controls ([30,31], reviewed in [32]), as well as modifications in genes implicated in lipid metabolism, have been observed in those populations [33]. This suggests that they may not only have higher TH levels but may also have a higher sensitivity to the effect of the hormone, at least with regards to its link between metabolism and heat production.

Another example linking TH with both heat production and resource availability is the hibernation strategy adopted by several animals, during which the decreased body temperature and complete absence of food consumption lead to a collapse of the metabolic rate. It is therefore not surprising to observe that hibernation is associated with a decrease of TH levels in bears, squirrels, hamsters, and woodchuck (reviewed in [11,34]). Recently, Frøbert *et al*. [35] found a 60% decrease of T4 and T3 levels in hibernating brown bears when compared to their active state. Hibernating bears are thus hypothyroid and share physiological features with hypothyroid humans such as decreased basal metabolic rate, bradycardia, and hypothermia.

The link between TH and thermogenesis also plays an important role in the thermal acclimation of ectotherms, that rely on external sources of heat for their body temperature, leading it to vary with the temperature of the environment. Warmer temperatures can induce higher metabolic rates, exceeding energy supplies and leading to oxidative stress; colder temperatures can notably reduce biochemical reaction rates and lead to higher reactive oxygen species concentrations, damaging DNA, proteins, and lipids [36]. It is therefore important for ectotherms to reduce energy expenditure in warm conditions, while they will need to upregulate biochemical pathways in cold conditions [36]. TH levels increase during cold exposure, enhancing metabolic rates and locomotor performance and facilitating adaptation to lower temperatures. Additionally, TH influence muscle function and cardiac performance, for instance by enabling zebrafish to maintain physiological activities despite temperature shifts [37,38]. Multiple studies have highlighted the role of the thyroid axis in the thermal acclimation of ectotherms, showing that it notably depends on T3 levels (e.g. in zebrafish; [38]) but also on peripheral regulators such as deiodinases ([36,39] and references therein). Similarly, the radiating *Barbus intermedius* of Lake Tana in Ethiopia [40] and, even more interestingly, the arctic charrs of Lake El'gygytgyn in the Russian Arctic have unique adaptation to cold and nutrient-poor environments and exhibit moderate thyroid status and reduced metabolic rate [41].

As TH are tightly interconnected with temperature and thermoregulation and play an important role in warm and cold adaptation, it will be crucial to fully understand the broader mechanisms of thermal acclimation in aquatic ectotherms to get insights into the impact of climate change on aquatic ecosystems.

## 4. Salinity acclimation

Many coastal fishes move between marine and freshwater habitats, which requires rapid physiological acclimation to changes in salinity. This acclimation is particularly important for estuarine fishes or fish that enter coastal lagoons, where they are exposed to highly variable environments. To successfully acclimate to different salinities, fish must efficiently control several physiological processes that allow them to maintain osmotic equilibrium. Numerous hormones are involved, notably prolactin, growth hormone, cortisol, and TH which will play an important role in salinity acclimation by stimulating ion-regulating tissues—such as gills, kidney, and intestine—to enhance ion uptake and secretion and maintain osmotic balance [42,43]. In parallel, endocrine pathways act to modulate the metabolic rate and energy expenditure, which can influence the overall metabolic cost of osmoregulation. Salinity acclimation further exemplifies how hormones orchestrate a suite of physiological adjustments crucial for organisms to thrive in diverse environments.

TH levels have been shown to be affected by changing salinities in several fish species, with the earliest studies dating back to the 1940s. Indeed, an early set of experiments showed an activation of TH in both freshwater fishes exposed to higher salinities and marine fishes exposed to lower salinities [44–46]. Euryhaline flounders and mummichogs (*Fundulus heteroclitus*) exposed to seawater had higher thyroid activity (in terms of iodine clearance) than freshwater-exposed ones [47,48]. More recent experiments confirm the involvement of TH in salinity acclimation. Freshwater climbing perch (*Anabas testudineus*) and grass carps placed at a high salinity underwent a transient increase in plasma T4 levels [49,50]. In addition, gilthead seabreams living in high salinity habitats and switching to low salinity habitats had higher *dio2* expression levels and higher free T4 and T3 levels than controls but, strikingly, did not show changes in TSH*β* levels. This suggests a peripheral, rather than central, effect on the HPT axis [51]. Overall, the TH pathway has been found to change when fish are exposed to different (higher or lower) salinities, hinting that it acts to allow fish to acclimate to new conditions.

TH have indeed been found to influence major osmoregulatory processes such as the activity of the sodium pump in the gills, as reported in euryhaline tilapias in freshwater where Na^+^/K^+^-ATPase activity increases in the gills and decreases in the kidneys upon T3 and T4 treatment [52]. Similarly, T4-treated marine flounders show strong Na^+^/K^+^-ATPase levels in mitochondria-rich gill cells [53]. Furthermore, there is evidence of interplay between TH and other osmoregulatory hormonal systems. For instance, T4 treatments were reported to enhance the ability of cortisol to stimulate branchial osmoregulatory function in tilapia, especially in saltwater-acclimated ones [54]. Similarly, an interaction between plasma cortisol and free T4 in the plasma was suggested to mediate the short-term response of sole *Solea senegalensis* exposed to higher salinities [55].

Additionally, genetic adaptation to environments with different salinities was recently observed in some euryhaline species. A study on sea bass found intraspecific differences in the physiological response to freshwater transfer suggesting a possible genetic adaptation, though the genes have not yet been identified [56]. Endocrine-related genetic differences were also shown in sea bream living in lagoon and marine locations, which display different profiles of nucleotide diversity in growth hormone and prolactin genes that may be an indication of genetic differentiation [57]. Interestingly, a metadata analysis of euryhaline fish populations living in different salinities found TR*α* to be a common selected gene [58]. This suggests that endocrine pathways, notably TH, participate to the osmoregulatory response in fishes over short time-periods (i.e. acclimation to the environment), but are also involved in salinity-related selective pressures driving adaptation over multi-generational timescales.

### (a) Seasonal adaptation

Metabolism, thermoregulation, and even adaptation to salinity variations are often linked to seasonal variations. TH play a critical role in the regulation of seasonality, the recurring patterns of biological changes that unfold cyclically throughout the year. Not only do TH intricately regulate functions like energy metabolism and body temperature (as described previously), but they also synchronize these processes with environmental fluctuations, such as the seasonal cycle with its photoperiod changes.

To anticipate annual environmental changes, vertebrates evolved an endogenous long-term timing mechanism, the circannual clock, that is synchronized based on the photoperiod through melatonin. In mammals, the *pars tuberalis* of the anterior pituitary is the central target of melatonin. It controls a photoperiod-induced increase of expression of the transcription factor Eya3 as well as TSH*β*, which act as key transducer of photic information to the hypothalamus, where subtle changes in local TH signalling activate the reproductive axis (see [59] for a review). This axis is directly controlled by a local increase of TH, resulting from a direct ‘retrograde’ effect of TSH*β* produced in the anterior pituitary without passing the HPT axis, regulating *dio2* and *dio3* expression levels [60]. This *dio2/dio3* balance is active in many vertebrates (mammals, birds, reptiles, amphibians, and fishes) with some changes in the anatomical site from one species to another but with a consistent impact of melatonin on TSH*β* in the anterior pituitary. Therefore, TSH*β* plays a central role by serving as the primary connection between photic cues and deiodinase expression changes.

Only a few studies have explored the connection of photoperiod and reproduction via TH in a natural setting. Bentley *et al*. [61] revealed seasonal variation in *dio2* but not *dio3* expression levels in wild European starlings (*Sturnus vulgaris*). Interestingly, starlings exhibit seasonal changes in singing behaviour and this plasticity has been linked, among other factors, to TH signalling [62,63]. Exciting prospects lie in investigating the connection between TH-driven seasonality and singing plasticity in this species.

Extreme environments also offer fascinating case studies to reveal constraints and biases acting on endocrine systems. This is the case for high latitude environments, with their extended periods of uninterrupted daylight in summer and night in winter. The Svalbard ptarmigan (*Lagopus muta hyperborea*) resides in such an environment [64]. Interestingly, despite the lost circadian control of behaviour in constant light conditions, this bird exhibits circadian-based photoperiodic timekeeping suggesting a different regulation of the retrograde TSH*β* regulation owing to the selective pressures of Arctic life.

A recent study focusing on year-round breeding goats detected five mutations in the *TSHβ* gene and a significant difference in genotype distributions between seasonal and non-seasonal breeds [65]. These results therefore suggest that *TSHβ* gene mutations could contribute to the diversity of reproductive seasonality in goats. A short sequence mutation in *TSHβ* in Yangzhou goose was also associated with reproduction [66]. In humans, data obtained on populations from Gambia demonstrated that methylation of *PAX8*, a gene critically important for thyroid gland development, is sensitive to the periconceptional environment, with rainy season conceptions having higher methylation compared to conceptions in the dry season [67]. In a follow-up study, the authors showed that *PAX8* hypomethylation at 2 years old is associated with a 21% increase in thyroid volume and an increase in free thyroxine (T4) at 5 to 8 years [68]. However, the link with the molecular mechanism controlling seasonality is still unknown.

### (b) Metamorphosis

As discussed in the previous sections, TH are key regulators allowing organisms to acclimate to a range of environmental conditions—temperature, salinity, seasons. This role in tailoring physiology to environmental conditions is crucial for metamorphosis, a process that is known to be controlled by TH in chordates [2]. Metamorphosis, the transformation of a larvae into a juvenile, is a key developmental step in numerous vertebrate taxa, but also invertebrate chordates like amphioxus [2,69]. It involves changes in internal and external body features, diet, and behaviour, which allow the organism to survive the transition from its larval to its juvenile environment. The classic example is the transformation of an aquatic tadpole to a terrestrial frog [70]. In fishes, especially marine fishes, larvae are typically planktonic, growing and dispersing in the open ocean. Metamorphosis enables juveniles to live in a different environment such as shallow coastal areas and reefs. Metamorphosis is thus inherently an ecological transformation associated with morphological, anatomical, and physiological changes. Importantly, this transformation is irreversible, making it a major commitment that hinges on reconciling environmental cues with internal conditions. The initiation of metamorphosis consistently is a balance between external and internal signals, with often conflicting environmental cues, which operate within the context of a developing organism.

Vertebrate metamorphosis is tightly controlled by each step of the TH cascade, from their production to their conversion by deiodinase, regulation of their nuclear receptor expression, and their subsequent tissue-specific effects on target gene expression. In amphibians, the timing of metamorphosis is controlled by the thyroid receptors TR*α* and TR*β*. First, in the absence of hormones, TR*α* blocks metamorphosis by recruiting corepressors [71] until a threshold in TH level is reached. At that time, TR*β* expression increases and is autoregulated through a positive feedback loop to reach high TH-sensitivity in tissues in conjunction to increases in TH levels ([72], reviewed in [73]). The peak in TR*β* autoregulation marks the onset of metamorphosis in numerous other taxa (e.g. amphibians; [72,74], fishes; [75]). As discussed above, other hormones (e.g. glucocorticoids) can inhibit or promote the action of TH for metamorphosis [76].

The TH cascade can be adaptively altered to modify metamorphosis in response to selective pressures (reviewed by [2]). Extreme cases such as direct development and paedomorphosis, where larvae reach sexual maturity, can be found in amphibians such as the tree frog *Eleutherodactylus coqui* (direct development), and in several salamanders (paedomorphosis) such as axolotls (*Ambystoma* spp.), mudpuppies *Necturus,* and their cave relative *Proteus* [77,78]. Interestingly, while TH treatment induces metamorphosis in *Ambystoma* spp., it has no visible effect on *Necturus* and *Proteus.* However, these cases are evolutionary modifications fixed in the genome. Amphibian metamorphosis can also be controlled by ecological factors, as illustrated by reduced larval duration in unfavourable conditions such as desiccating ponds, increased temperatures, or limited food availability (reviewed in [6,79,80]).

Drying ponds obviously greatly impact the survival of tadpoles [81,82]. The common response to drying ponds is to accelerate the larval/tadpole development and the timing of metamorphosis [82–84]. This early metamorphosis is an adaptive response that allows the individuals to escape deteriorating environmental conditions by transforming into a terrestrial organism. However, this rapid development comes at a price. For instance, the spadefoot toad species, *Pelobates cultripes*, normally has a long larval duration; however, when metamorphosis is accelerated by low water levels, it leads to a smaller (metamorphosed) body size with shorter limbs, and increased oxidative stress [83,85]. Furthermore, it was shown that early metamorphosed individuals have a more homogeneous, less complex pigmentation as adults, an effect that is not yet understood [86]. Spadefoot toads with a normally long tadpole phase (e.g. *Pe. cultripes*) can greatly reduce its duration under unfavourable environmental conditions, whereas species with usually short phases (e.g. *Scaphiopus couchii*) seem to have lost this developmental plasticity. Strikingly, the adult morphology of the short-developing *Sc. couchii* is more compact with shorter limbs, similarly to the larvae of more plastic species in drying ponds [84,85].

**Figure 3.**
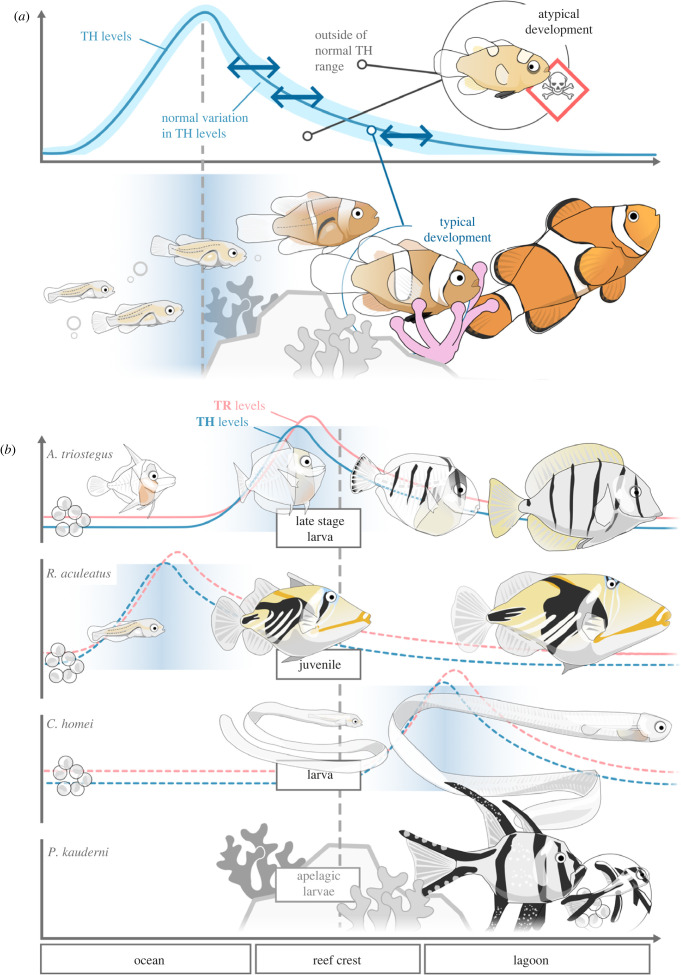
TH levels coordinate the metamorphosis of marine fishes with their life history and ecological transitions. (*a*) A peak in TH levels is associated with the metamorphosis of pelagic fish larvae, often as they transition from the open ocean to reefs and coastal environments. While a natural range of TH levels is conducive to proper development and successful metamorphosis, large deviations in either direction lead to developmental defects and larvae/juveniles maladapted to their new environment. (*b*) Shifts in the maturity of coral reef fish entering the reef habitat and implication for the peak of TH and TR expression with respect to their life history. In the surgeonfish *Acanthurus triostegus*, the falling limb of the peak in TH and TR expression coincides with the crossing of the reef crest, timing metamorphosis with the imminent ecological change. In other species, different life stages are seen crossing the reef: already metamorphosized juveniles in the case of *Rhinecanthus aculeatus*, or non-metamorphosed larvae for the carapid *Carapus homei*. Other species, such as *Pterapogon kauderni* complete their entire lifecycle within the lagoon, and therefore do not have pelagic stage at all: they are considered as ‘apelagic’ species and may be reminiscent of direct developing frogs.

The evolutionary and ecological variations of metamorphosis timing in fishes remain largely unexplored. Coral reef fishes, with their unique life cycle, offer potential insights into metamorphosis plasticity in natural settings. Most species lay pelagic eggs, and after hatching, the larvae grow as part of the open ocean plankton for several weeks, benefiting from reduced predation and the opportunity for dispersal [87,88]. When the larvae are ready to settle on a reef, their metamorphosis is triggered [2,88]. Studies on surgeonfish have shown that the peak of TH is initiated early on, while the larvae are still in the open ocean, and that the entry in the reef corresponds to the falling limb of the TH peak [21,89,90] (figure 3). The triggers initiating metamorphosis are still not understood. However, the wide intraspecific variation observed in pelagic larval duration (PLD) implies a regulation that connects ecological conditions with TH, corticoids levels, and metamorphosis. Environmental conditions can influence TH signalling, potentially affecting the quality of juveniles [89,90] and their local adaptation to the habitat in which they settle as juveniles [91]. Developmental plasticity and phenotypic disparity in response to environmental diversity highlight the acclimation of fishes to their local environment during metamorphosis. The role of hormonal variation in these situations undoubtedly requires further investigation, and there are a multitude of cases that will be of interest for future studies.

As with the PLD, there are great interspecific variations in TH levels and changes in the fish as they enter the reef [89]. For example, a study comparing surgeonfish with a butterflyfish, a triggerfish, an apogonid and a damselfish, showed differences in the rate and magnitude of the TH level changes that accompany the entry in the reef [89] (figure 3). Some species (*Pterapogon kauderni*, *Acanthochromis*) have an apelagic life-history reminiscent of the direct development in frogs (figure 3). While TH appears to be instrumental in larval recruitment to the reef, these variations illustrate potential species-specific modifications revealing that not all species enter the reef at the same level of maturity. Clearly, this post-embryonic developmental stage is influenced by evolution and varies based on species-specific life history characteristics. Considering their remarkable array of life history strategies, coral reef fishes offer a valuable framework to uncover the triggers of these changes and the role of hormonal system modifications.

Metamorphosing larvae experience a multitude of challenges, like feeding from patchy resources, maturing their sensory systems, and avoiding predators. Such challenges imply that a very fine regulation of metabolic processes should occur during metamorphosis, so that larvae can have enough energy to face these highly demanding processes. With the effects of TH we described so far, it is no surprise that TH impact the activity of lipid metabolism enzymes, therefore influencing energy availability in the transforming larvae [92]. Furthermore, transcriptomic analysis of larval development in several fish species have revealed differences in transcript levels of genes involved in metabolic pathways, namely glycolysis, the citric acid cycle, and fatty acid β-oxidation [21,93]. In anemonefish, metamorphosis marks the shift between anaerobic and aerobic metabolism and TH are involved in the regulation of this transition [21]. Clearly, more studies shedding light on how TH control metabolism during metamorphosis would be particularly desirable to fully reconcile the action of TH in triggering and coordinating metamorphosis and its known metabolic function.

## 5. Conclusion

All of the considerations above point to a common function of TH shared by vertebrates and, probably, by most phyla of bilateral animals [2,94–96]: ensuring that metabolism and more generally energy expenditure are consistent with the environment, i.e. harmonizing animal function with its ecosystem. TH have been found to vary in numerous taxa, notably seasonally, and with food availability, day length, temperature, and growth. Given the selection for adaptable metabolic strategies to enhance fitness, TH play a pivotal role in finely tuned phenotypically plastic strategies that anticipate and adapt to environmental shifts across seasons and changing conditions.

However, it must be made clear that focusing on TH alone could be rightly viewed as too simplistic to account for environment-mediated metabolic adjustments. Changes in corticosteroids and/or other hormonal pathways (such as appetite-controlling molecules, e.g. leptin) probably accompany changes in TH. These hormones are not measured in most studies, despite being major regulators of metabolism and energy expenditure (e.g. [97,98]). Deciphering how major hormonal pathways collectively fine-tune these regulations is an important direction for future studies.

**Figure 4.**
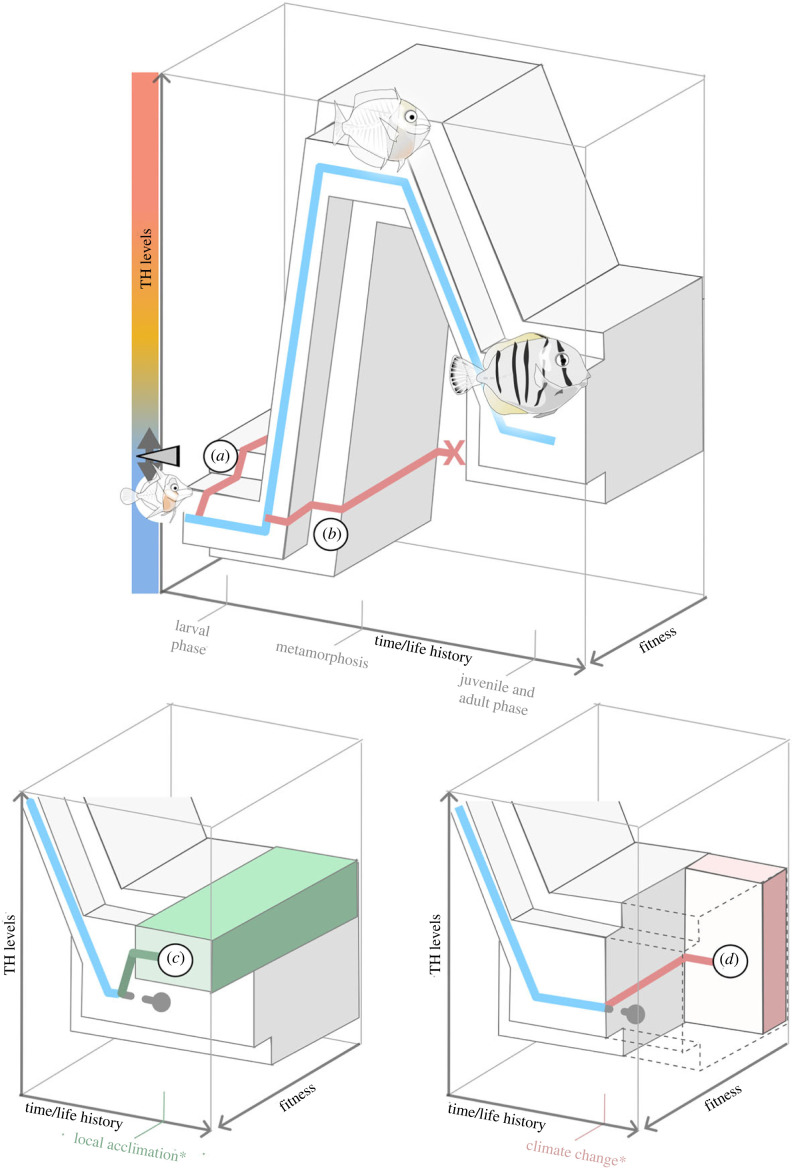
A general model for the ecological role of TH. Organisms (here, a developing surgeonfish) can maintain optimal fitness while navigating ontogenetic and ecological changes, with often drastically different physiological demands, by tuning their TH levels. Blue path: optimal progression in TH levels throughout early development (here, metamorphosis). (*a*) Detuning between the organism and the environment owing to precocious changes in TH levels. (*b*) Detuning between the organism and the environment owing to a failure in changing the TH levels (e.g. owing to endocrine disruptors in the environment). (*c*) Local acclimation: the organism fine tunes its TH levels (here, slight increase) to better match its environment and thus improve its fitness. The thick dotted grey line indicates maintenance of suboptimal fitness levels in the absence of TH tuning. (*d*) Detuning between the organism and the environment owing to a sudden change in environmental conditions (e.g. climate change). The thick dotted grey line indicates maintenance of high fitness at the given TH level in the absence of ecological changes. Events marked with an asterisk are illustrated as happening at the juvenile and adult stages, but may occur at any timepoint throughout the life cycle.

It is also crucial to recognize that TH do not solely serve this regulatory role in adults but are also a major player during post-embryonic development, notably as coordinators of metamorphosis, notably in coral reef fishes (figure 4). During this period, TH ensure metabolic coordination, the transformation of organs, and body growth via their effects on the skeleton [99,100]. TH control the speed and magnitude of the transformation so that these energetically costly changes do not exceed what the environment can provide. This is even more important since the larva will change its ecological niche by entering a new habitat to which it has to adapt as a juvenile. The metabolic and developmental function of TH must therefore be combined to allow the harmonious transformation of the larva into a juvenile, developing within a dynamic and complex environment. We must acknowledge, however, that this view is a model that remains to be supported by clear experimental data. Future studies will confirm whether the impact of TH on metabolic pathways is consistent with expectations of how these pathways should change to assist the ontogenetic transition in matching the environment. Work is in progress in our laboratory and elsewhere to provide such data.

This brings us to a last important notion: the idea that TH allow the transformation from larva to juvenile to occur in harmony with the surrounding environment is of course linked to the notion of local adaptation. We believe that, in each generation, TH are essential players in the local acclimation of juveniles resulting from metamorphosis to their local environment and our work on anemonefish and surgeonfish goes in this direction [21,90]. This view is supported by data suggesting the acclimation and adaptation of animal populations via the modulation of the TH pathway (for example: [17,18]). In particular, TH changes also occur at the evolutionary scale, allowing true local adaptation of animals. This line of research should now be a priority if we want to better understand the ecological function of TH.

This is also important because multiple human activities induce harmful effects in animals and in particular on the youngest life stages. In fact, many anthropogenic stressors and pollutants [101], artificial light at night [102,103], and climate change [90,104] act by disrupting TH signalling pathway at one level or another. We believe that in many cases these stressors actually induce disharmonies which produce animals that are not truly adapted to their environment, leading to a reduced ecological quality and little chance of survival (figure 4). Similar causes may be implicated in the ‘carry-over’ effects that were described in several contexts including human diseases [105–107]. This general model of the ecological function of TH provides a useful framework that can be tested and challenged.

## Data Availability

This article has no additional data.
